# A Resonant Pressure Microsensor with the Measurement Range of 1 MPa Based on Sensitivities Balanced Dual Resonators

**DOI:** 10.3390/s19102272

**Published:** 2019-05-16

**Authors:** Yulan Lu, Pengcheng Yan, Chao Xiang, Deyong Chen, Junbo Wang, Bo Xie, Jian Chen

**Affiliations:** 1 State Key Laboratory of Transducer Technology, Institute of Electronics, Chinese Academy of Sciences, Beijing 100190, China; luyulan15@mails.ucas.ac.cn (Y.L.); yanpengcheng17@mails.ucas.ac.cn (P.Y.); xiangchao115@mails.ucas.ac.cn (C.X.); chenjian@mail.ie.ac.cn (J.C.); 2 University of Chinese Academy of Sciences, Beijing 100049, China

**Keywords:** resonant pressure microsensor, 1 MPa, dual resonators, sensitivity

## Abstract

This paper presents a resonant pressure microsensor with the measurement range of 1 MPa suitable for the soaring demands of industrial gas pressure calibration equipment. The proposed microsensor consists of an SOI layer as a sensing element and a glass cap for vacuum packaging. The sensing elements include a pressure-sensitive diaphragm and two resonators embedded in the diaphragm by anchor structures. The resonators are excited by a convenient Lorentz force and detected by electromagnetic induction, which can maintain high signal outputs. In operation, the pressure under measurement bends the pressure-sensitive diaphragm of the microsensor, producing frequency shifts of the two underlining resonators. The microsensor structures were designed and optimized using finite element analyses and a 4” SOI wafer was employed in fabrications, which requires only one photolithographic step. Experimental results indicate that the Q-factors of the resonators are higher than 25,000 with a differential temperature sensitivity of 0.22 Hz/°C, pressure sensitivities of 6.6 Hz/kPa, and −6.5 Hz/kPa, which match the simulation results of differential temperature sensitivity of 0.2 Hz/°C and pressure sensitivities of ±6.5 Hz/kPa. In addition, characterizations based on a closed-loop manner indicate that the presented sensor demonstrates low fitting errors within 0.01% FS, high accuracy of 0.01% FS in the pressure range of 20 kPa to 1 MPa and temperature range of −55 to 85 °C, and the long-term stability within 0.01% FS in a 156-day period under the room temperature.

## 1. Introduction

Pressure microsensors are widely used in the fields of industrial metering, aerospace aviation, meteorology, medical, automobiles, and consumer electronics [1,2,3,4], which function according to several typical working principles, such as capacitive sensing [5,6], piezoresistive sensing [7,8], piezoelectric sensing [9,10], fiber optical sensing [11,12], and resonant sensing [13,14]. Compared to other types of pressure microsensors, resonant pressure microsensors are featured with high resolutions, high accuracy, long-term stability, and quasi-digital outputs [15,16], which meet the urgent demands of gas pressure calibration equipment in the field of industrial process control.

The resonant pressure microsensor was first introduced by Greenwood in 1984, and is based on electrostatic excitation/capacitive detection [17]. However, the resonator was not packaged in a vacuum, and the microcapacity is hard to detect due to the unwanted interference capacitance, unless in a vacuum environment below 133 Pa. In 1991, Peterson et al. introduced a resonant pressure microsensor based on silicon fusion bonding [18] which achieved vacuum packaged for the resonator. The Q-factor of this sensor was higher than 100,000 and the accuracies were 0.01% FS (Full Scale) in the temperature range of −40 to 125 °C within the pressure measurement range of 10 to 130 kPa. Nevertheless, the vibration directions of the two resonators mentioned above are perpendicular to the pressure-sensitive diaphragm, which would result in energy losses. A laterally driven micromachined resonant pressure sensor was introduced by Welham et al. in 1996 [19]. This sensor uses a hammock resonator as a sensing element and the Q-factor is insensitive to the leakage of cavity gases due to the laterally driven principle. Due to the benefits of being laterally driven, Sun et al. presented a micromachined resonant pressure sensor based on an electrostatic excited/capacitance detected resonator in 2016 [20]. Experiment results showed that the sensor had a pressure sensitivity of ~29 Hz/kPa, a nonlinearity of 0.02% FS, a hysteresis error of 0.05% FS within the pressure measurement range of 20 to 280 kPa, and the temperature sensitivity of the resonator was ~2 Hz/°C in the temperature range of −40 to 80 °C. Meanwhile, Du introduced a resonant pressure microsensor based on an electrostatic excitation/capacitive detection resonator which showed a pressure sensitivity of −8.7 Hz/kPa, a maximum error of 0.0310% FS under the pressure measurement range of 30to 190 kPa and temperature range of 30 to 70 °C [21]. However, those developed sensors included only one resonator which might cause the output frequency strongly sensitive to temperature. Our previous works developed several types of resonant pressure sensors based on dual resonators, such as Luo (2014) [22], Xie (2015) [23], and Shi (2018) [24], whereas the lowest working temperature was constrained, which mainly result from the mismatched sensitivities of two resonators. In addition, the pressure measurement ranges of the developed sensor mentioned above were less than 300 kPa, and much larger pressure measure ranges had not been reported. The compromised pressure measurement range might result from the nonlinearity of frequency responses to the pressure under measurement.

To address these issues, this study presents a resonant pressure microsensor based on dual resonators using convenient magnetic excitation/magnetic detection. This sensor was designed based on balanced temperature and pressure sensitivities within the linear elastic range to explore the low working temperature extremity and enlarge the pressure measurement range. The frequency responses of the resonant sensor were analyzed based on finite element analysis (FEA) simulations. The resonant pressure microsensor was fabricated and experimental characterizations including open-loop tests, closed-loop tests, and long-term tests to validate the design.

## 2. Design

### 2.1. Working Principle

**Figure 1:**
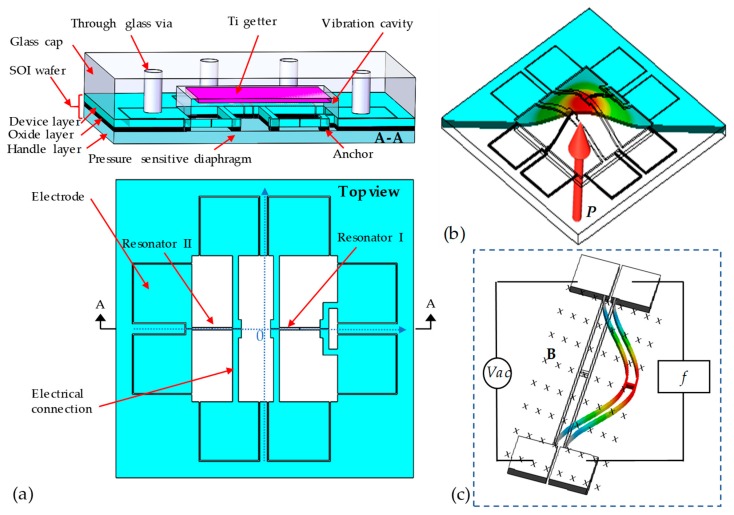
Schematic of the proposed resonant pressure microsensor: (**a**) The proposed pressure microsensor consists of an SOI wafer (including a pressure-sensitive diaphragm, two H-shaped resonators, and eight electrode pads) and a glass cap (including a vibration cavity with getter inside and eight through-glass vias); (**b**) Details of the sensing elements when pressure applied. The pressure-sensitive diaphragm bends under the pressure, which changes the axial stresses of the resonators; (**c**) The resonator is excited based on electromagnetic forces and detected based on electromagnetic inductions.

The proposed resonant pressure microsensor is shown in Figure 1. The sensor chip with a size of 10 × 10 × 0.842 mm^3^ consists of a glass cap with eight vias (Φ 600 μm) with a cavity (5 mm × 5 mm × 120 μm) and an SOI (Silicon-on-insulator) wafer with sensing elements. The sensing elements include a pressure-sensitive diaphragm (5 mm × 5 mm × 300 μm) in the handle layer of the SOI wafer, and two H-shaped resonators (two single 1400 μm × 18 μm × 40 μm beams with a connection of 60 μm × 18 μm × 40 μm) in the device layer in the SOI wafer. The doubly clamped H-shaped resonators, which are deployed on the central (resonator I) and border (resonator II) areas of the diaphragm, respectively, are coupled to the diaphragm by anchor structures in the oxide layer of the SOI wafer (see Figure 1a).

In operations, the deflections of the pressure diaphragm build up stresses when the pressure under measurement is applied (see Figure 1b). In this study, the resonator I and resonator II are located in the tensile and compressive area of the diaphragm, which leads to the resonant frequency shifts of resonator I increasing, and those of resonator II decreasing. Note that, the two resonators exhibit the same temperature properties for the same materials and fabrication processes, which means that the differential frequency outputs are immune from temperature variances. For the frequency readout, a permanent magnet was used to provide a uniform magnetic field. An ac signal is fed into one beam of the H-shaped resonator, and the resonator driven by electromagnetic force. On the other hand, the other beam of the resonator can pick up the resonant frequency signals based on the principle of electromagnetic induction (see Figure 1c).

### 2.2. FEA Simulations

FEA simulations based on ANSYS software were employed to design and optimize the balanced pressure and temperature sensitivities within the linear elastic range of the resonant pressure microsensor. Multimodels of static structural and modal were used to calculate the intrinsic frequency shifts in response to pressure under measurement variances, and multimodels of steady-state thermal, static structural, and modal were used to calculate the intrinsic frequency shifts in response to surrounding temperature variances. In the simulations, tetrahedral elements were used to mesh the geometrical structures of the microsensor with ~350,000 elements. 

The boundary conditions of pressure sensitivity simulations were defined that the edges of the pressure-sensitive diaphragm were constrained to avoid unconstrained movements. In the static structural part, pressures of 100 kPa to 1 MPa were used as the loads. The generated stress distributions within the structures were then used as the loads for the modal part, and the outputs of the simulations were the intrinsic frequency shifts of the resonators in response to the pressure applied.

The initial conditions of temperature sensitivity simulations were set using the reference temperature as 350 °C, which is the bonding temperature of the glass cap and the SOI wafer. In addition, a reference pressure of 100 kPa was introduced in the static structure part for the consideration of convenient comparison to experimental results. In the steady-state thermal simulations, temperatures of −55 to 85 °C were used as the loads and the temperature distributions of the whole structure were transferred to the static structural part. The calculated stresses within the structures were then used as the loads for the modal part, and the outputs of the simulations were the intrinsic frequencies of the resonators in response to temperature variances.

**Figure 2:**
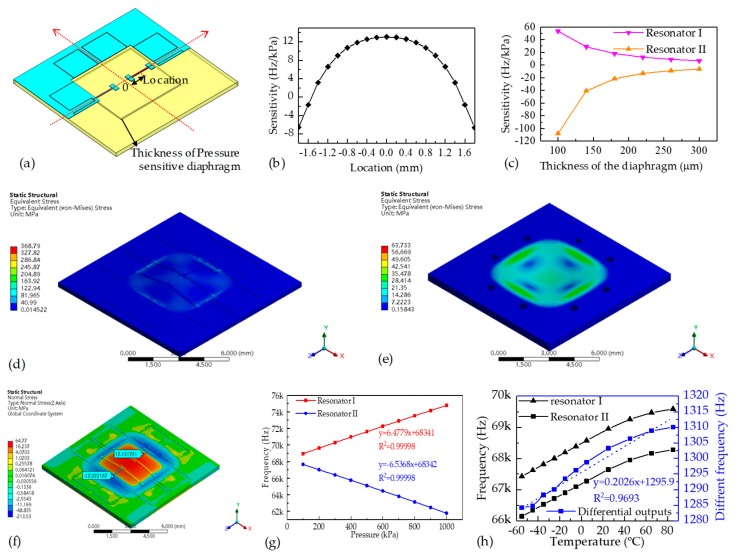
(**a**) The established geometry using in simulations; (**b**) The sensitivity variances in response to (**b**) resonator locations and (**c**) the thickness of the pressure-sensitive diaphragm. The equivalent stress distributions in (**d**) the SOI layer and (**e**) in the glass cap with a pressure of 1 MPa applied. (**f**) The normal stress distributions along the axial direction of the resonator with a pressure of 1 MPa applied. (**g**) The intrinsic frequency responses to applied pressure. (**h**) The intrinsic frequency responses to applied temperature.

Figure 2a shows the geometry built in the FEA simulations. Figure 2b shows the sensitivity variance in response to the locations of the resonators where the thickness of the pressure-sensitive diaphragm was 300 μm. Figure 2c shows the sensitivity variances in response to the thickness of the pressure-sensitive diaphragm when resonator I was located at +1.2 mm and resonator II was located at −1.8 mm from the center of the pressure-sensitive diaphragm. 

From the results of the resonator location simulations and the diaphragm thickness simulations, it can be found that the pressure sensitivities of the microsensor are well-matched when resonator I was positioned at +1.2 mm and resonator II was positioned at −1.8 mm from the center of the pressure-sensitive diaphragm when the thickness of the pressure-sensitive diaphragm was 300 μm. The balanced pressure sensitivities would contribute to temperature compensations due to inversed pressure-sensing properties and similar temperature-sensing properties, which could further extend the pressure measurement range and working temperature range.

Figure 2d–f represent the stress distributions in the developed microsensor under an applied pressure of 1 MPa. The equivalent stresses in the SOI wafer are within the yield strength of 7 GPa (see Figure 2d) and the stresses in the glass cap are within the yield strength of 69 MPa (see Figure 2e), which shows the safety of the microsensor structures. In addition, tensile/compressive stresses are induced in resonator I/II with the values of ±17.1 MPa (see Figure 2f), which indicate that the frequency shifts of the two resonators are the same, but in reversed directions. 

Furthermore, the intrinsic frequency shifts as a function of applied pressure and surrounding temperature are shown in Figure 2g,h. The pressure sensitivities of the two resonators were quantified with ±6.5 Hz/kPa and linear coefficients of 0.9999 in the pressure range of 20 kPa to 1 MPa and a reference temperature of 22 °C, and the temperature sensitivity was quantified as 0.2 Hz/°C in the temperature range of −55 to 85 °C with a reference pressure of 100 kPa. 

## 3. Fabrication

**Figure 3:**
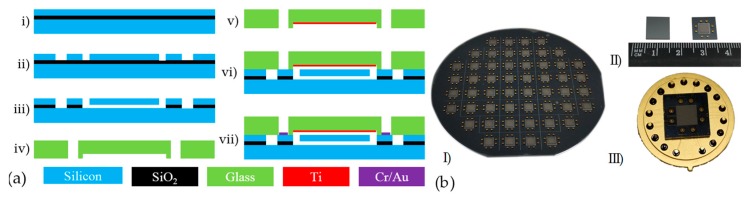
(**a**) The fabrication processes: (i) Cleaning the SOI wafer; (ii) Forming the sensing elements; (iii) Releasing the resonators; (iv) Forming the through-glass vias and cavity in glass wafer; (v) Sputtering Ti as getter; (vi) Anodic bonding; (vii) Cr/Au metallization. (**b**) The fabrication results: (I) Wafer after metallization; (II) Sensor chips after dicing; (III) Packaging prototype.

Conventional and simplified SOI–MEMS (Micro-Electro-Mechanical System) fabrication processes, which include only one photolithography step, were used to fabricate the proposed resonant pressure microsensor as shown in Figure 3. A 4” SOI wafer ((100) plane, <100> oriented, p-type, device layer of 40 μm, handle layer of 300 μm, an oxide layer of 2 μm) and a 4” BF33 glass wafer with a thickness of 500 μm were employed in device fabrications. The main fabrications include deep reactive ion etching, HF releasing, and anodic bonding.

The SOI wafer was immersed in deionized water and dried with pure N_2_ gas after being cleaned by piranha etchant to remove the organic molecules and boiled deionized water to remove soluble ions (see Figure 3a(i)). Then, using patterned photoresist as a mask, the exposed device layer of the SOI wafer was etched through to form the resonators (see Figure 3a(ii)). Note that the handle layer of the SOI wafer remains unetched to form a pressure-sensitive diaphragm with a thickness of 300 μm. After that, the underlying SiO_2_ layer of the resonators was removed by immersing the SOI wafer in an HF solution in a time-controlled manner (see Figure 3a(iii)). 

The glass wafer was cleaned using the same processes of the SOI wafer. The through-glass vias for electrical connections and the cavities for housing the vibration of the resonators in the BF33 glass wafer were drilled by laser in a program-controlled manner (see Figure 3a(iv)). Then, Ti was sputtered on the cavity as the getter material for gas absorption during next anodic bonding process (see Figure 3a(v)). 

After finishing the fabrications of the SOI and the glass wafers, anodic bonding was utilized to form vacuum encapsulations for resonators where the voltage, and tool pressure and temperature of anodic bonding were set at 600 V, 1000 mbar, and 350 °C, respectively (see Figure 3a(vi)). Then, Cr/Au (200 Å/1000 Å) films were sputtered on the through-glass vias to form electrical connections by using a hard mask (see Figure 3a(vii)). The fabrication results were shown in Figure 3b, including the microsensor wafer after Cr/Au metallization (see Figure 3b(I)), the front/back views of the microsensor chips after dicing (see Figure 3b(II)), and the microsensor after packaging (see Figure 3b(III)).

## 4. Experimental Characterizations

**Figure 4:**
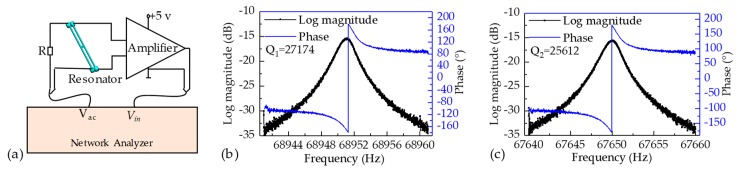
(**a**) Schematic of the open-loop measurements for the resonators of the proposed microsensor. The intrinsic frequencies, phase shifts, and Q-factors of resonator I (**b**) and resonator II (**c**) under an atmospheric pressure of ~100 kPa and room temperature of ~25 °C.

The open-loop circuit was used to quantify the intrinsic frequencies, phase shifts, and Q-factors of the two resonators of the proposed microsensor under an atmospheric pressure of ~100 kPa and room temperature of ~25 °C as shown in Figure 4a. A network analyzer was used to supply an ac voltage to one beam of the resonator and pick up the voltage induced by the other beam of the resonator. The intrinsic frequency of the resonator I was quantified as 68.951 kHz with the phase shift of ~180° and Q-factor of 27174 (see Figure 4b). Besides, the intrinsic frequency of the resonator II was quantified as 67.650 kHz with the phase shift of ~180° and Q-factor of 25612 (see Figure 4c). The mismatches of the Q-factors mainly result from the different structural damping effects of the two resonator structures.

**Figure 5:**
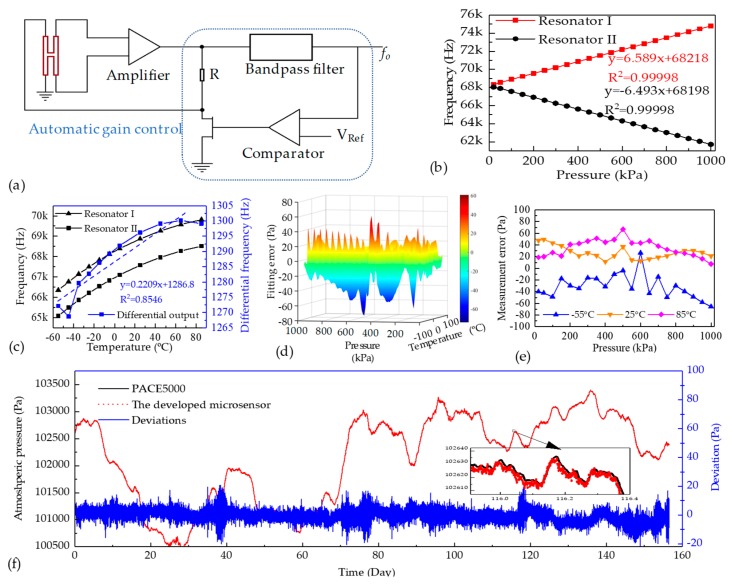
(**a**) Schematic of the closed-loop circuit. (**b**) The frequency responses as functions of the applied pressure in room temperature. (**c**) The frequency responses as functions of the surrounding temperature with the reference pressure of 100 kPa. (**d**) The fitting errors of the developed microsensor within the full pressure range of 20 kPa to 1 MPa and temperature range of −55 to 85 °C. (**e**) The measurement errors to the standard pressure of 20 kPa to 1 MPa under the surrounding temperature of −55, 25, and 85 °C. (**f**) The comparisons of the developed microsensor and the PACE5000 pressure calibrator in actual atmospheric pressure measurement.

In order to further characterize the performances of the proposed microsensor, a closed-loop circuit producing the self-oscillation signal was developed, as shown in Figure 5a. An amplifier was used to amplify the voltage induced by the vibrations of the resonator, and the amplified voltage was then depressed and sent to the driving beam of the resonator for exciting the resonator in a closed-loop manner. In order to maintain the stable vibrations of the resonator, an automatic gain- controlled module, including a bandpass filter, a comparator, and a field effect transistor, was introduced in the closed-loop circuit. 

A pressure calibrator (PPC4, FLUCK) and a temperature chamber (SU-262, ESPEC) were employed to provide the pressure under measurement and surrounding temperature during the characterization processes. In this study, the microsensor was characterized within a pressure range from 20 kPa to 1 MPa and a temperature range from −55 to 85 °C.

Figure 5b shows the intrinsic frequencies of the two resonators as a function of pressure under measurement at 25 °C, producing the pressure sensitivities and linearly dependent coefficient of +6.6 Hz/kPa, 0.99998 for resonator I and −6.5 Hz/kPa, 0.99998 for the resonator II. Figure 5c shows the intrinsic frequencies of the two resonators as a function of surrounding temperature with a pressure of 100 kPa applied, producing the differential temperature sensitivity of 0.22 Hz/°C in the temperature range of −55 to 85 °C. The two results validated the accuracies of the FEA simulations. 

Considering the wide working temperature range of the developed microsensor, temperature compensation was performed in this study. As mentioned above, the frequency of the resonators can be expressed as functions of pressure and temperature, which means that the pressure *P* can be obtained by the frequencies of resonators (*f*_1_ and *f*_2_) based on mathematical translation [25]. Then, the pressure *P* can be expressed as

(1) $$P=a_{0}+a_{1}f_{1}+a_{2}f_{2}+a_{3}f_{1}{}^{2}+a_{4}f_{1}f_{2}+a_{5}f_{2}{}^{2}+a_{6}f_{1}{}^{3}+a_{7}f_{1}{}^{2}f_{2}+a_{8}f_{1}f_{2}{}^{2}+a_{9}f_{2}{}^{3},$$

where *a*_0_, …, *a*_9_ are temperature compensation coefficients.

Figure 5d shows the fitting errors of the proposed microsensor in the full pressure and temperature ranges following the custom temperature compensation algorithm, producing the compensation errors within ±76 Pa with corresponding ±0.01% FS, which indicates that the developed microsensor is stable enough under the heat conditions from −55 to 85 °C. Figure 5e shows the measurement errors of the microsensor under the surrounding temperatures of −55, 25, and 85 °C, which demonstrates a high accuracy with quantified measurement errors within ±67 Pa with the corresponding ±0.01% FS. These results validate the functionalities of the balanced pressure sensitivities in extending the pressure and temperature ranges.

Furthermore, the long-term measurements for the actual atmospheric pressure of the developed microsensor in room temperature were conducted for a 156-day period. As shown in Figure 5f, the pressure deviations between the developed microsensor and the PACE5000 Pressure Calibrator (GE Druck, USA) are less than ±20 Pa with the corresponding ±0.002% FS. These results validated the stabilities of the resonant pressure microsensor.

Table 1 shows the comparison of the proposed microsensor to previously reported counterparts, which indicated the proposed microsensor demonstrate higher pressure measurement range, lower working temperature extremity, lower temperature sensitivity, equivalent Q-factor, and equivalent accuracy.

## 5. Conclusions

A resonant pressure microsensor with the measurement range of 1 MPa was presented, where FEA simulations were used to design the sensor structure. The microsensor was fabricated based on conventional and simplified fabrication processes, which need only one photolithography step. The experimental results show that the sensitivities of the two resonators of the developed sensor are 6.6 Hz/kPa and −6.5 Hz/kPa, both with linearly dependent coefficients of 0.99998, which match the simulation results. The differential temperature sensitivity of the developed sensor is 0.22 Hz/°C, which is a benefit resulting from the design of the dual resonator. Moreover, the Q-factors of the two resonators were quantified at higher than 25,000. Further characterizations based on a closed-loop manner indicate that the developed sensor demonstrates low fitting errors within 0.01% FS, and low measurement errors within 0.01% FS under the pressure range of 20 kPa to 1 MPa in a temperature range of −55 to 85 °C. The long-term stability of the proposed microsensor was validated by comparing the measurements of the actual atmospheric pressure with PACE5000 calibrator in a 156-day period, exhibiting a lower deviation within 0.002% FS.

## Figures and Tables

**Table 1:** Comparisons of microsensor performances.

Reference	Q-Factor	Pressure Range	Temperature Range	Temperature Sensitivity	Accuracy
Peterson [18]	>100,000	10~130 kPa	−40~125 °C	~2 Hz/°C	0.01% FS
Welham [19]	50 in air	0~350 kPa	-	-	-
Sun [20]	10,000	20~280 kPa	−40~80 °C	2 Hz/°C	0.05% FS
Du [21]	20,000	30~190 kPa	30~75 °C	-	0.03% FS
Luo [22]	22,000	50~100 kPa	−40~70 °C	−0.33 Hz/°C	0.02% FS
Xie [23]	11,000	50~110 kPa	−40~70 °C	-	0.02% FS
Shi [24]	10,000	10~150 kPa	−35~85 °C	−0.30 Hz/°C	0.01% FS
This sensor	>25,000	20~1000 kPa	−55~85 °C	+0.22 Hz/°C	0.01% FS
